# Development and Fabrication of a Multi-Layer Planar Solar Light Absorber Achieving High Absorptivity and Ultra-Wideband Response from Visible Light to Infrared

**DOI:** 10.3390/nano14110930

**Published:** 2024-05-25

**Authors:** Cheng-Fu Yang, Chih-Hsuan Wang, Pei-Xiu Ke, Teen-Hang Meen, Kuei-Kuei Lai

**Affiliations:** 1 Department of Chemical and Materials Engineering, National University of Kaohsiung, Kaohsiung 811, Taiwan; cfyang@nuk.edu.tw (C.-F.Y.); a1105609@mail.nuk.edu.tw (C.-H.W.); m1115615@mail.nuk.edu.tw (P.-X.K.); 2 Department of Aeronautical Engineering, Chaoyang University of Technology, Taichung 413, Taiwan; 3 Department of Electronic Engineering, National Formosa University, Yunlin 632, Taiwan; 4 Department of Business Administration, Chaoyang University of Technology, Taichung 413, Taiwan

**Keywords:** multi-layer, planar, solar light, absorber, evaporation technique, focused ion beam (FIB), transmission electron microscopy (TEM)

## Abstract

The objective of this study is to create a planar solar light absorber that exhibits exceptional absorption characteristics spanning from visible light to infrared across an ultra-wide spectral range. The eight layered structures of the absorber, from top to bottom, consisted of Al_2_O_3_, Ti, Al_2_O_3_, Ti, Al_2_O_3_, Ni, Al_2_O_3_, and Al. The COMSOL Multiphysics^®^ simulation software (version 6.0) was utilized to construct the absorber model and perform simulation analyses. The first significant finding of this study is that as compared to absorbers featuring seven-layered structures (excluding the top Al_2_O_3_ layer) or using TiO_2_ or SiO_2_ layers as substituted for Al_2_O_3_ layer, the presence of the top Al_2_O_3_ layer demonstrated superior anti-reflection properties. Another noteworthy finding was that the top Al_2_O_3_ layer provided better impedance matching compared to scenarios where it was absent or replaced with TiO_2_ or SiO_2_ layers, enhancing the absorber’s overall efficiency. Consequently, across the ultra-wideband spectrum spanning 350 to 1970 nm, the average absorptivity reached an impressive 96.76%. One significant novelty of this study was the utilization of various top-layer materials to assess the absorption and reflection spectra, along with the optical-impedance-matching properties of the designed absorber. Another notable contribution was the successful implementation of evaporation techniques for depositing and manufacturing this optimized absorber. A further innovation involved the use of transmission electron microscopy to observe the thickness of each deposition layer. Subsequently, the simulated and calculated absorption spectra of solar energy across the AM1.5 spectrum for both the designed and fabricated absorbers were compared, demonstrating a match between the measured and simulated results.

## 1. Introduction

Since Landy et al. initially introduced the concept of nearly perfect absorbers in 2008 [1], there has been a growing interest in exploring these absorbers over the past decade. This heightened interest is largely due to their significant relevance in various fields such as solar cells [2], sensors [3,4], and numerous other applications [3,4,5]. The advancements in metamaterial absorbers have primarily followed distinct trends tailored to specific applications. These trends encompass achieving narrower ideal absorption bands for enhanced sensing capabilities [6,7,8], creating multi-band absorbers for achieving perfect absorption across multiple frequencies [8,9,10,11], and enhancing absorptivity within the solar band for efficient solar power generation [12,13,14]. There are many mechanisms that can be used to investigate the absorbers with high absorptivity and ultra-wideband properties. Consequently, a plethora of studies have been conducted to explore various technologies aimed at constructing absorbers with exceptionally high absorptivity across an ultra-wideband (UWB) spectrum. This ongoing research and development endeavors are driven by the desire to harness the full potential of nearly perfect absorbers in diverse technological contexts. For example, Cui et al. showcased a significant advancement in the field with the introduction of an infrared absorber made from saw-toothed anisotropic metamaterial [15]. This inventive design allowed the absorber to achieve a remarkable absorbance of more than 90% across a broad spectrum ranging from 3000 nm to 5000 nm.

Absorber photon-trapping layers are specifically designed structures or materials aimed at maximizing light absorption within a photovoltaic device, such as a solar cell. These layers are crucial for enhancing the efficiency of solar energy conversion by increasing the amount of sunlight absorbed by the active semiconductor material [16,17,18]. The primary function of absorber photon-trapping layers is to confine and manipulate light within the active region of the solar cell, thereby increasing the optical path length and the probability of photon absorption. By trapping photons within the absorber layer for longer periods, more of the incident sunlight can be absorbed, leading to higher conversion efficiencies. Frequency-selective absorbers are indeed crucial structures in sensing applications, especially in areas like spectroscopy, imaging, and remote sensing. These absorbers are designed to selectively absorb or reflect light at specific frequencies while allowing other frequencies to pass through or remain unaffected. This selective absorption makes them valuable for detecting and analyzing specific wavelengths of light, which can carry important information about the environment, materials, or substances being studied. Danila et al. conducted simulations to analyze the spectral characteristics of non-layered gold-silicon and all-silicon frequency-selective metasurfaces with an asymmetric element configuration within the mid-infrared spectrum, specifically ranging from 5 to 5.8 μm. Their findings suggest that the metasurface architecture they proposed shows promise as a potential candidate for various applications in mid-infrared technology, including absorbers, sensors, and imaging systems [19]. Jeong et al. highlighted recent advancements in the development of reconfigurable metasurfaces utilizing advanced materials. These metasurfaces are designed for frequency-selective absorption across a wide range of spectra, including the visible, terahertz, sub-terahertz, millimeter-wave, and microwave bands [20].

In a similar vein, Ding et al. introduced an ultra-wideband (UWB) polarization-independent metamaterial absorber. Their design featured a periodic arrangement of multilayered quadrangular frustum pyramids, each with distinct side lengths tailored to different absorption modes. This approach demonstrated the versatility and potential of metamaterials in achieving broadband absorption across a wide range of frequencies [21]. The planar optical absorbers, relative to other absorber designs, have certain advantages in certain situations [22,23]. These advantages include:

(a) Planar optical absorbers are very compact and occupy minimal space, and that makes them suitable for limited installation spaces and easy integration into various devices and systems.

(b) The planar optical absorbers is typically relatively straightforward and can be achieved using simple nanofabrication techniques. This makes large-scale production easier and allows for cost control.

(c) Due to their compactness and ease of manufacturing, the planar optical absorbers are more easily integrated into various optical and optoelectronic systems, including solar cells, optical communication systems, optical sensors, and more.

(d) The design of the planar absorbers can be adjusted and optimized according to specific application requirements to achieve the desired performance. This flexibility makes them suitable for a wide range of different application scenarios.

Therefore, the multilayer structures composed of different materials can be utilized to vary the electromagnetic properties to achieve broadband absorption. Using impedance matching as a design strategy for high absorptivity and ultra-wideband absorbers also offers the advantages of improved energy transfer, reduced reflection, broadband performance, and consistency. In this study, we aimed to identify the ideal structural parameters for a designed absorber with high absorptivity characteristics across an ultra-wideband spectrum spanning from near-ultraviolet to visible light and the near-infrared (NIR) range. To achieve this goal, we conducted numerical simulations and analyses on a multilayer thin-film structure. It is worth noting that previous research efforts have also investigated the accuracy and reliability of numerical analysis results obtained using COMSOL Multiphysics^®^ [24,25]. Therefore, the commercially available COMSOL Multiphysics^®^ simulation software was utilized for constructing the simulation models and processing the simulations. Several mechanisms are employed in the design of absorbers with high absorptivity and an ultra-wideband capability. One key approach is the utilization of multilayer stack design, which involves the creation of layered structures using various materials possessing diverse electromagnetic properties. This strategy is harnessed to achieve extensive absorption across a broad spectrum of frequencies. To optimize this process, careful consideration is given to adjusting layer thicknesses and their arrangement, ultimately maximizing absorption efficiency across a wide frequency spectrum. Another critical element in absorber design is impedance matching. This technique is employed to configure the absorber structure so that its impedance aligns with that of the surrounding environment, including free space, over a wide range of frequencies.

This alignment is essential for ensuring the efficient transfer of energy from incident waves to the absorber. This research presents several significant findings in the context of absorber optimization. The first noteworthy observation pertains to the performance of absorbers featuring eight-layered structures, particularly when compared to counterparts lacking the top Al_2_O_3_ layer or utilizing TiO_2_ or SiO_2_ layers as substitutes for Al_2_O_3_. When designing planar-type light absorbers, several challenges may arise. Firstly, there is a limitation in light absorption efficiency due to the confined propagation path of light within the planar absorber, thereby restricting its absorption capability. Additionally, light reflection at the surface of the absorber can cause losses. Furthermore, optical losses such as scattering and absorption may occur during the propagation of light within the absorber. To overcome these issues, methods such as optimizing the thickness of the light absorption layer and designing appropriate optical structures can be employed. Utilizing an eight-layer structure as the primary design aims to enhance the performance of this optical absorber in solar energy harvesting applications. This choice is primarily motivated by the constructive interference of light waves between different layers in the planar structure. This eight-layer configuration is expected to exhibit excellent absorption characteristics across various wavelengths in the solar spectrum and effectively convert light energy into heat.

**Figure 1:**
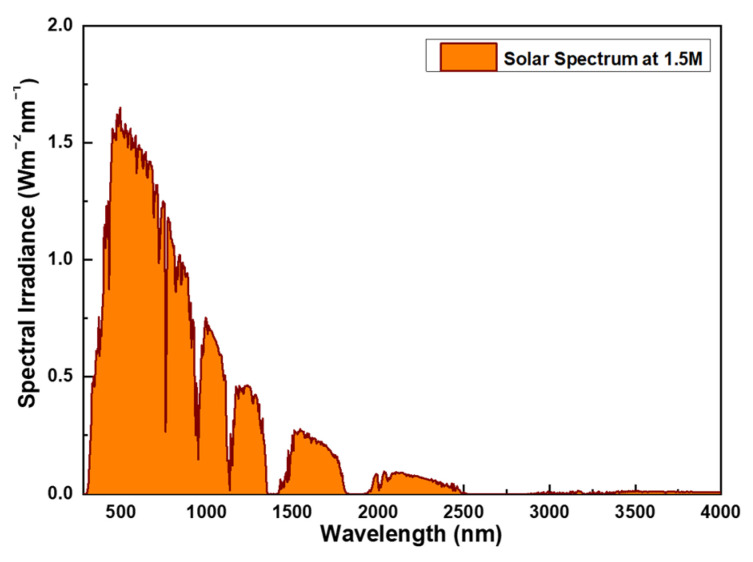
The spectrum of the AM1.5 solar irradiation.

Understanding the relationship between solar energy and wavelength is fundamental to the design of solar absorbers. The solar spectrum covers a wide range from ultraviolet to infrared, with varying energy and intensity corresponding to each wavelength, as depicted in Figure 1 [26]. The energy distribution of the solar spectrum primarily consists of:

(1) Ultraviolet (UV) range: with a predominant wavelength distribution between 340–400 nm, corresponding to photon energies ranging approximately from 3.1 eV to 124 eV, constituting less than 10% of solar energy.

(2) Visible Light range: primarily spanning from 400–700 nm, with photon energies ranging approximately from 1.77 eV to 3.1 eV, representing about 40% of solar energy. This segment of the spectrum is crucial for processes like photosynthesis in plants and the efficiency of photovoltaic cells.

(3) Infrared (IR): spanning from 700 nm to 1 mm, commonly applied within the range of 700–1900 nm as illustrated in Figure 1. Near Infrared (NIR) photon energies range approximately from 0.5 eV to 1.77 eV, with even lower energies in the far-infrared. Infrared energy constitutes over 50% of solar energy.

From this, it is evident that for a broadband absorber, if the absorption spectrum spans from 350–1900 nm, it can absorb over 95% of solar energy. Physical vapor deposition (PVD) is one of the commonly used methods for fabricating nano-scale optical thin films, especially for films requiring high quality, uniformity, and precise control over thickness and structure [27]. This approach utilizes physical processes such as sputtering, evaporation, or laser ablation to deposit raw materials in the form of atoms or molecules onto the surface of a substrate, forming thin films. Some advantages of physical vapor deposition include high purity and uniformity, precise control over film structure and thickness, applicability to various materials (including metals, oxides, nitrides, etc.), and lower residual stress. Therefore, in this study, we employed furnace vapor deposition, a method within physical vapor deposition, to deposit the multi-layer absorbers we designed.

It was found that the inclusion of the top Al_2_O_3_ layer yielded the most favorable anti-reflection effect, suggesting a critical role in enhancing overall absorber efficiency. A second intriguing revelation stems from the investigation into impedance-matching effects. The top Al_2_O_3_ layer demonstrated superior impedance-matching performance and significantly augmenting the absorptivity in comparison to scenarios where it was absent or replaced by TiO_2_ or SiO_2_ layers. This dual functionality proved to be instrumental in achieving an impressive average absorptivity of 96.76% across an ultra-wideband range spanning 350 to 1970 nm. The third significant aspect of this study involved the determination of optimal parameters for each layer, followed by the implementation of the evaporation method for the deposition and manufacturing of the designed absorber. Subsequent thorough measurements underscored the alignment between measurement results and simulation outcomes, validating the robustness and reliability of the proposed absorber design. The final focus of this study is on the multi-layer absorber fabricated using focused ion beam (FIB) cutting. After the fabrication, transmission electron microscopy (TEM) was employed to observe the deposited thin films on each layer. By utilizing the observed thickness for simulations, we found that the simulated absorption spectra based on TEM-observed thickness closely matched the results obtained from actual measurements.

Different top-layer materials were employed to investigate the absorption and reflection spectra, as well as the optical-impedance-matching properties of the designed absorber. Structural analyses of the fabricated multi-layer planar solar light absorber were conducted through FESEM observation, AFM analysis, and TEM observation. In addition, the study compared various data sets: the initial set results derived from simulation using optimal parameters (the COMSOL simulation data), the second and third sets measured from two different locations on identical samples of the fabricated multi-layer planar solar light absorber, and the fourth set integrating thickness data acquired from TEM observation into COMSOL simulations. Furthermore, the study compared the simulated and calculated absorption of solar energy by the designed and fabricated absorbers across the AM1.5 spectrum. These comparisons provide valuable insights into the performance and effectiveness of the fabricated absorbers in harvesting solar energy.

## 2. Structure Design, Simulation Process, and the Fabrication, Analyses, and Measurements of the Designed Absorber

**Figure 2:**
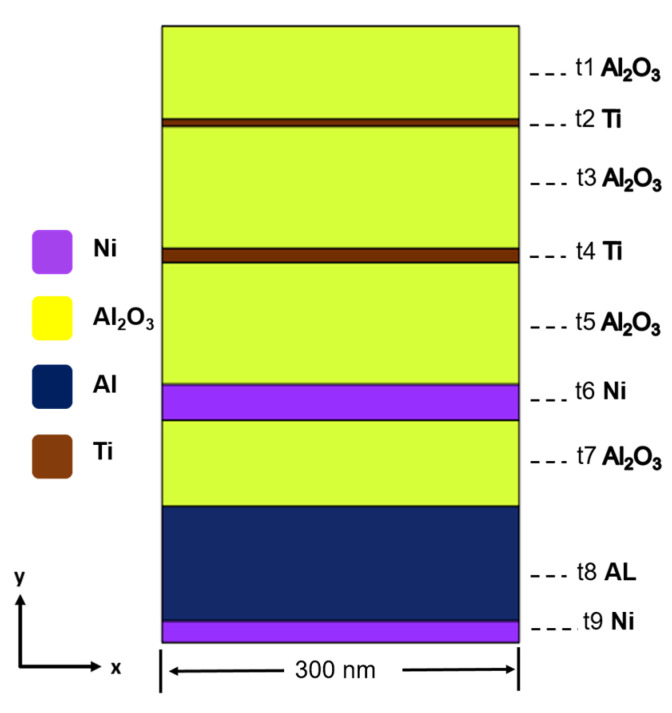
Schematic diagram of the designed ultra-wideband and high-absorptivity absorber.

The objective was to pinpoint the optimal thickness for each layer, thus guaranteeing that the designed absorber attains peak absorption efficiency across the specified wavelength range under investigation. To ascertain the most effective thickness for each layer, a methodical layer-by-layer approach was employed. In this process, the thickness of each material layer was systematically adjusted from the top to the bottom, while maintaining the thickness of the other layers constant. Utilizing this methodology to seek thickness optimization within the wavelength range of 300 to 2500 nm, even when employing the same materials, variations in thickness can lead to distinct absorption rates. This meticulous layer-wise adjustment of thickness allows for fine-tuning the absorber’s performance to match specific wavelength requirements and achieve superior absorption characteristics across a broad spectrum. Figure 2 provides a visual representation of the two-dimensional profile of a specially engineered ultra-wideband absorber with exceptionally high absorptivity. This absorber was comprised of multiple layers, each serving a unique purpose in enhancing its performance. Starting from the top and progressing downwards, the absorber structure consisted of the eight continuous planar layers.

The proposed absorber consisted of a top Al_2_O_3_ layer (t1), an upper Ti layer (t2), an upper middle Al_2_O_3_ layer (t3), a lower Ti layer (t4), a lower middle Al_2_O_3_ layer (t5), a metal Ni layer (t6), a lower Al_2_O_3_ layer (t7), and an Al substrate layer (t8). The Al layer provided a sturdy foundation for the absorber, the Ni and both Ti layers served as an integral component for absorbing electromagnetic radiation and optimized and enhanced the absorber’s absorption characteristics, and the top Al_2_O_3_ was used as the anti-reflection and optical layer. To verify whether the top Al_2_O_3_ layer could effectively integrate with the underlying structure to provide better impedance matching, we compared simulation results using an Al_2_O_3_ layer as the top layer with those without an Al_2_O_3_ layer as the top layer, or using TiO_2_ or SiO_2_ layers instead as the top layer. We aimed to demonstrate that employing an Al_2_O_3_ layer as the top layer could enhance the absorber’s overall efficiency compared to scenarios where no top oxide layer was used, or where TiO_2_ or SiO_2_ layers were utilized as top oxide layer. After the optimal thickness of each layer was found, a tube furnace vapor deposition machine was used to deposit the designed multilayer films on a Si/Ni substrate from the bottom to the top.

Si wafer was utilized as the substrate, and to enhance the adhesion of the Al_2_O_3_ thin film, a layer of approximately 15 nm thick nickel (Ni) metal was initially deposited onto the Si substrate. Additionally, to confirm that the presence of Ni thin metal and Al substrate did not impact the overall structural characteristics, both components were included in the optimized design of the 8-layer structure, resulting in a 10-layer configuration for analyses. It was observed that the analysis results of the 10-layer structure with the inclusion of Ni thin metal and Al substrate showed no difference from the optimized design of the 8-layer structure. The absorber under investigation boasts a sophisticated mesh structure with 780 grid nodes, with a maximum and a minimum grid lengths of 2.0 nm and 1.0 nm, and designed to accommodate incident radiation wavelengths between 300 nm and 3000 nm. Complementing these features are 254 finite elements, 1478 triangular elements, 20 endpoint elements, and 254 edge finite elements, contributing to its comprehensive design. Furthermore, the mesh demonstrates remarkable quality metrics, with an average element quality of 0.8183 and a minimum element quality of 0.5641. Notably, the mesh’s element/area ratio, calculated at 160,500 nm^2^, ensures the precision and reliability of the simulation results, affirming its suitability for rigorous analysis.

To demonstrate that the extra two layers have no effect on the overall structure, we conducted a comparative analysis. We compared the performance of absorptivity of the structure with and without the extra two layers. The absorptivity spectrum of the structure with the two extra layers was similar to that of the structure without the extra layers, therefore, it would indicate that the extra layers had no significant effect on the overall performance. Additionally, we may have conducted sensitivity analyses by varying the thicknesses related to the two extra layers to further validate that they do not significantly alter the performance of the structure. By comparing the simulation results and conducting sensitivity thicknesses’ analyses, we can provide evidence to support the conclusion that the extra two layers have no substantial effect on the overall structure. Consequently, for subsequent practical measurements, the effects of Ni thin metal and Al substrate will be disregarded.

The production processes were as follows: 1. Ultrasonic cleaning of the substrate, 2. drying, 3. mounting the workpiece on hangers, 4. placing it into the vacuum chamber, 5. inputting coating layer settings, and 6. starting the equipment for the coating process. When the coating process began, the vacuum in the chamber was initially reduced to 8.0 × 10^−6^ torr, and during the coating process, the coating vacuum was maintained at 4.0–8.0 × 10^−4^ torr. The coating temperature was approximately 40 degrees Celsius, and the coating power was 10 kW. After the multilayer films were deposited, scanning electron microscopy (FESEM) was used to observe the surface morphology of the topmost t1 Al_2_O_3_ layer. Atomic force microscopy (AFM) played a crucial role in assessing the planarity and smoothness of the topmost t1 Al_2_O_3_ layer, ensuring precise measurements. Focused ion beam (FIB) and transmission electron microscopy (TEM) are both powerful techniques used in materials science and nanotechnology for imaging the internal structures of materials. At first, FIB was used to prepare the sample and TEM was employed for surface morphology analysis, providing detailed insights into the topography of the thin films. To delve further into the optical properties of these thin films, we conducted optical measurements using a UV/visible/near-infrared spectrophotometer, specifically the HITACHI U4100 (Tokyo, Japan). This instrument allowed us to examine the absorption characteristics of the thin films within a wavelength range spanning from 300 to 2600 nm. This wavelength range was carefully chosen as it encompasses the critical region where thin film absorbance is of particular interest. By combining these advanced analytical techniques, we were able to gain a comprehensive understanding of the thin films’ physical and optical properties, contributing valuable insights to our research.

## 3. Results

**Figure 3:**
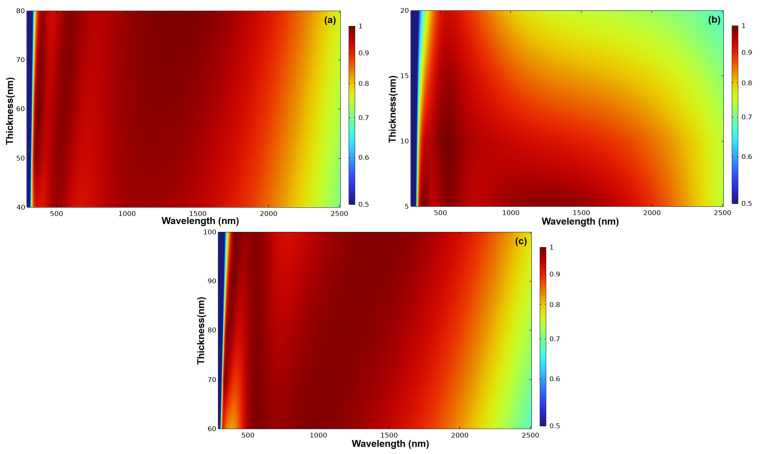
Influences of different thicknesses of each layer on the absorptivity of the designed multilayer absorber (**a**) top Al_2_O_3_ (t1) layer, (**b**) upper Ti (t2) layer, and (**c**) upper middle Al_2_O_3_ (t3) layer.

Figure 3 provides a comprehensive overview of how changes in the thickness of a single layer impact the absorptivity of the absorber design, while keeping all other layers unchanged, as initially depicted in Figure 2. The optimization process involved determining the ideal thickness within the wavelength range of 300 to 2500 nm. Interestingly, even when using the same material, it was necessary to vary the thicknesses for simulation. The results depicted in Figure 3 clearly illustrate the significant impact of modifying the thicknesses of different layers on the numerical fluctuations in absorptivity. In Figure 3a, we specifically examine the alterations in absorptivity brought about by adjusting the thickness of the Al_2_O_3_ anti-reflection layer (t1) within the range of 40 to 80 nm, while maintaining the overall structure presented in Figure 2, with t2 = 5 nm, t3 = 85 nm, t4 = 10 nm, t5 = 85 nm, t6 = 25 nm, t7 = 60 nm, and t8 = 100 nm. As observed in Figure 3a, increasing the thickness of the t1 layer from 40 nm to 80 nm led to a noteworthy enhancement in absorptivity, exceeding 90%, and shifted the absorptivity range from approximately 330–2000 to about 350–2280 nm. This outcome strongly implies that an increase in the thickness of the t1 layer results in a shift of the lower and upper bounds for achieving absorptivity greater than 0.9 towards higher wavelengths.

When the thickness of the top Al_2_O_3_ layer (t1) was about 65 nm, the designed absorber had a high absorptivity and exhibited the best absorption efficiency in the range of 350–2280 nm. Therefore, the final choice for the thickness of Al_2_O_3_ layer (t1) was 65 nm, as in the final thickness simulation result. To find the optimal thickness for Ti layer (t2), an analysis of simulated parameters was conducted in the range of 300 to 2500 nm, with Ti layer (t2) thickness varying from 5 to 20 nm. According to the results shown in Figure 3b, it is evident that below a thickness of 10 nm, there was a predominance of the red region for wavelengths ranging from about 350 to 2200 nm, indicating better absorption in this region. However, as the wavelength approached 200 nm, there was an increasing presence of the blue region near the 20 nm thickness, suggesting a decrease in absorption. Therefore, the final choice for the thickness of Ti layer (t2) is 5 nm, as it exhibited the optimal absorption efficiency in the thickness analysis simulation of the Ti layer (t2). Our simulations have shown that our designed photodetector exhibits outstanding absorption properties when the Ti layer (t2) is 5 nm thick. In this study, we aim to compare the performance of our device through both experimental and simulated approaches. It is worth noting that choosing a thickness below 5 nm could introduce deviations in the experimental results. Therefore, to ensure the reliability and accuracy of our experimental findings, we have opted for a 5 nm thickness for the t2 layer.

Figure 3c illustrates the variation of absorptivity of the designed absorber caused by only changing the thickness of the upper Al_2_O_3_ layer (t3) from 60 to 100 nm. According to Figure 3c, when the wavelength of light was around 100 nm, the region where the blue absorption was poor became more prominent as the thickness became closer to 100 nm. Additionally, in the wavelength range of approximately 250–400 nm, absorptivity was lower for thicknesses between 60–80 nm. As the thickness increased, the range of blue absorption shifted towards longer wavelengths (redshift). Therefore, the final choice for the thickness of the upper Al_2_O_3_ layer (t3) was 85 nm, as it exhibited the optimal absorption efficiency in the thickness analysis of this layer. The variations of absorptivity of the designed absorber caused by only changing the thicknesses of the lower Ti layer (t4, changed from 5 to 20 nm), the lower middle Al_2_O_3_ layer (t5, changed from 60 to 100 nm), the metal Ni layer (t6, changed from 10 to 30 nm), the lower Al_2_O_3_ layer (t7, changed from 40 to 80 nm), and the Al metal layer (t8, changed from 80 to 120 nm) and the simulation results were not shown here.

When the thicknesses of the t4, t5, t6, t7, and t8 layers were set to 10 nm, 85 nm, 25 nm, 60 nm, and 100 nm, respectively, the designed absorber exhibited high absorptivity in the range of approximately 350 to 2000 nm. Therefore, we selected thicknesses of 65 nm, 5 nm, 85 nm, 10 nm, 85 nm, 25 nm, 60 nm, and 100 nm as the optimized thicknesses for the top Al_2_O_3_ layer (t1), upper Ti layer (t2), upper middle Al_2_O_3_ layer (t3), lower Ti layer (t4), lower middle Al_2_O_3_ layer (t5), metal Ni layer (t6), lower Al_2_O_3_ layer (t7), and Al layer (t8), respectively. The substrate Al layer in this study serves two primary functions. First, it acts as a bottom reflector layer to enhance absorption efficiency. Second, it serves as a stabilizing layer at the base, simplifying the fabrication process. This is why a thickness of 300 nm is directly employed. According to the simulation results, when the thickness of the Al layer varies between 80 and 300 nm, there is no significant change in the overall absorption efficiency within the wavelength range of 300 to 2500 nm.

**Figure 4:**
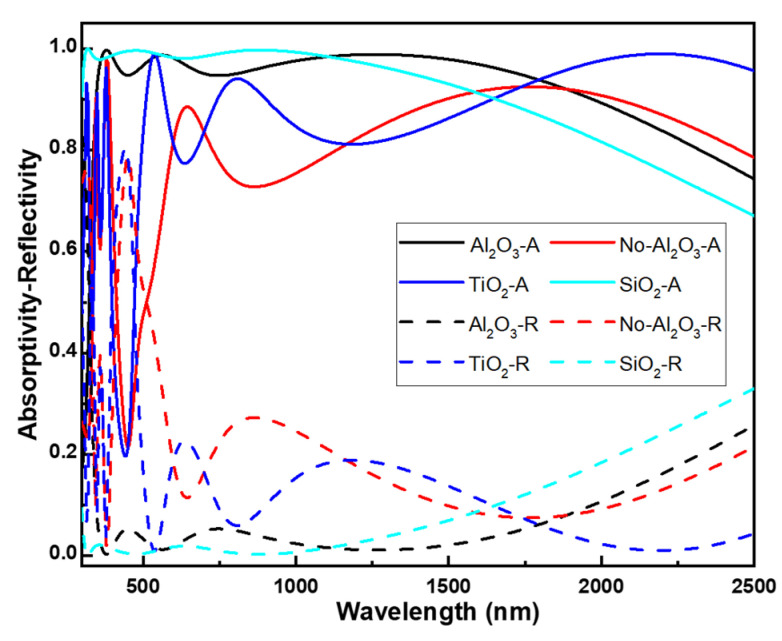
Effects of different top-layer materials on the absorption and reflection spectra of the designed absorber. A: absorption spectrum, R: reflection spectrum.

Figure 4 displays the numerically simulated absorption and reflection spectra of the designed absorber under normal incident light. The equation A + R + T = 1 is commonly used to describe the interaction of light with a surface or material, where A represents the absorptivity of the incident light, i.e., the fraction of incident light that is absorbed by the material; R represents the reflectivity of the incident light, i.e., the fraction of incident light that is reflected off the surface of the incident material; T represents the transmissivity of the incident light, i.e., the fraction of incident light that passes through the material. In this context, the equation indicates that the total energy of the incident light is conserved. When light strikes a material, some of it may be absorbed, some may be reflected, and the rest may pass through the material. The total of these three fractions (A—absorptivity, R—reflectivity, and T—transmissivity) sums up to 1, signifying the complete energy of the incident light is accounted for. In order to demonstrate the superior anti-reflection capabilities of the top Al_2_O_3_ layer, we examined the absorption and reflection spectra of three distinct structures: the designed eight-layer planar thin-film structure (depicted in Figure 2), a seven-layer planar thin-film structure (without top Al_2_O_3_ layer), and the designed eight-layer planar thin-film structure with substitutions of TiO_2_ or SiO_2_ for the top Al_2_O_3_ layer, and all the simulation results are compared in Figure 4. Notably, the simulated transmissivity for all mentioned absorbers within the wavelength range of 300–2500 nm is uniformly zero (not presented here). Figure 4 also illustrates that the combined absorptivity and reflectivity sums to 1 in all 4 mentioned conditions.

When comparing the spectral characteristics of structures with and without Al_2_O_3_ anti-reflective layers, a clear pattern emerges. Structures lacking Al_2_O_3_ anti-reflective layer exhibit notably reduced absorptivity in the 300–1950 nm range compared to their Al_2_O_3_-layered counterparts. However, a closer inspection reveals a significant nuance: for wavelengths below approximately 680 nm, the absorptivity spectrum of Al_2_O_3_-absent structures displays pronounced variations. These observations suggest that Al_2_O_3_ plays a multifaceted role beyond its anti-reflective properties, contributing to an enhanced overall absorptivity spectrum. When the alternative top anti-reflective layer is explored, such as TiO_2_ with matching thickness, we find that the absorptivity within the 400–1800 nm range is lower than that of structures employing Al_2_O_3_ as the top anti-reflective layer. However, when SiO_2_ is used with the same thickness as the top anti-reflective layer, an interesting phenomenon occurs. Although the low-wavelength high absorptivity (absorptivity higher that 90%) range shifts to approximately 310 nm, the high-wavelength high absorptivity range shifts to around 1610 nm. These results demonstrate that Al_2_O_3_ layer exhibits the optimal anti-reflective effect and once again confirm that, in addition to its anti-reflective properties, there are other factors that make the use of Al_2_O_3_ layer as the anti-reflective coating in this study’s absorber design result in optimal absorptivity characteristics. Figure 4 illustrates that while the reflectivity of SiO_2_ is lower than that of Al_2_O_3_ within the range of 350–1100 nm, in the absence of an Al_2_O_3_ layer or when TiO_2_ and SiO_2_ layers are used as the topmost layer in multilayer structures, the average reflectivity of these three conditions surpasses that of Al_2_O_3_ within our analyzed range. Consequently, the average absorptivity is noticeably poorer when Al_2_O_3_ is not used as the topmost layer or when TiO_2_ or SiO_2_ layers replace Al_2_O_3_. The reasons behind this discrepancy will be elucidated in the analysis of optical impedance. This observation underscores the significant impact of material choice on the optical properties of multilayer structures, with TiO_2_ and SiO_2_ exhibiting distinct reflective behaviors compared to Al_2_O_3_.

Impedance matching refers to the most suitable pairing between a signal source or transmission line and a load. It ensures the most efficient transfer of signal power from the source to the load, minimizing reflections as much as possible during the transmission process. This becomes particularly prominent in the context of light propagation in metallic structures and related metamaterials. Impedance of light is a physical quantity that describes a material’s response to optical waves, typically used to study a material’s optical properties at different wavelengths or frequencies. The real part of optical impedance is the material’s impedance to optical waves and is also known as the real part of refractive index. It represents the phase change and reflection characteristics of light when propagating through the material. The real part of optical impedance is often used to describe the material’s refractive effect on light. When the real part of optical impedance equals 1 (Z-real = 1), it typically implies that light waves propagate through the material without undergoing a phase change, meaning they are not refracted or reflected but propagate at the same speed.

The imaginary part of optical impedance is usually employed to describe the material’s absorption behavior towards optical waves and provides information about the absorption or scattering of photon energy by the material. When the imaginary part of optical impedance equals zero, it indicates that the material exhibits minimal absorption of light. In the field of optics, this situation is referred to as optical transparency. A zero imaginary part of optical impedance implies that light waves propagating through the material are not absorbed and almost entirely penetrate the material. The relationship between anti-reflection efficiency and optical impedance matching is profound, with optical impedance matching being a crucial concept for maximizing anti-reflection efficiency. Anti-reflection efficiency quantifies the ability of a surface or material to reduce reflection and optical impedance, on the other hand, delves into the intricacies of the electrical resistance and capacitance properties that light encounters as it propagates through materials or interfaces. It is typically linked to properties such as refractive index, material thickness, and surface characteristics.

Optical impedance matching, therefore, refers to the adjustment of material properties to minimize or eliminate reflection at the interface between a material and its surrounding medium. This typically involves the careful selection of specific materials and the design of layer thicknesses to ensure that the optical impedance of the anti-reflection layer matches that of the surrounding medium. When optical impedance is matched in this way, reflection is minimized, resulting in the maximization of anti-reflection efficiency. In summary, the intricate interplay between anti-reflection efficiency and optical impedance matching underscores the significance of deliberately choosing and designing materials to achieve optimal anti-reflective performance. This concept holds great importance in various optical applications, including absorbers, solar cells, lens coatings, and optical filters, where minimizing reflection and enhancing optical system performance are paramount.

**Figure 5:**
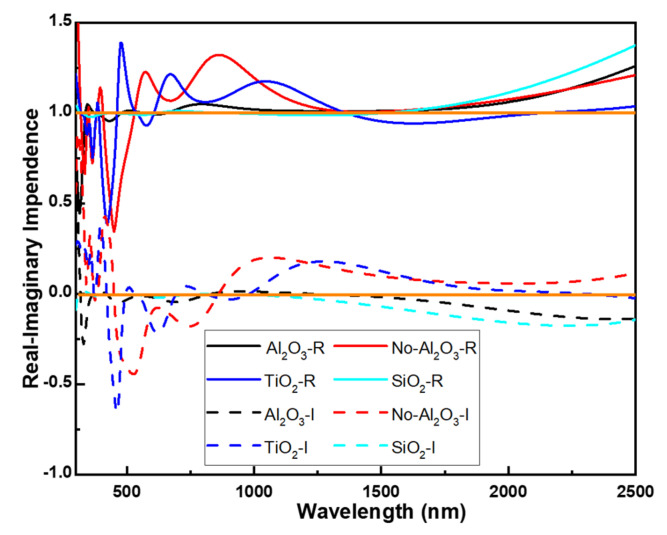
Effects of different top-layer materials on the real and imaginary spectra of the designed absorber. R: real impendence, I: imaginary impendence.

We believe this represents the primary factor contributing to the development of a multi-layer planar solar light absorber with exceptional characteristics, including high absorptivity and ultra-wideband functionality. The analysis of optical impedance clearly demonstrates that in the absence of an Al_2_O_3_ layer or when TiO_2_ or SiO_2_ layers replace Al_2_O_3_, the real parts of optical impedance for these 3 conditions deviate from a value of 1 earlier compared to when Al_2_O_3_ is used, as revealed by the optical impedance analysis, as Figure 5 shows. Similarly, the imaginary parts also exhibit an earlier departure from a value of 0 for all 3 conditions compared to when Al_2_O_3_ is utilized. This suggests that the substitution of Al_2_O_3_ with TiO_2_ or SiO_2_ layers affects the optical properties of the structure, causing deviations in both the real and imaginary parts of the optical impedance. These deviations likely stem from differences in the refractive indices and thicknesses of the materials used, indicating the importance of material selection in optimizing the optical performance of multilayer structures.

**Figure 6:**
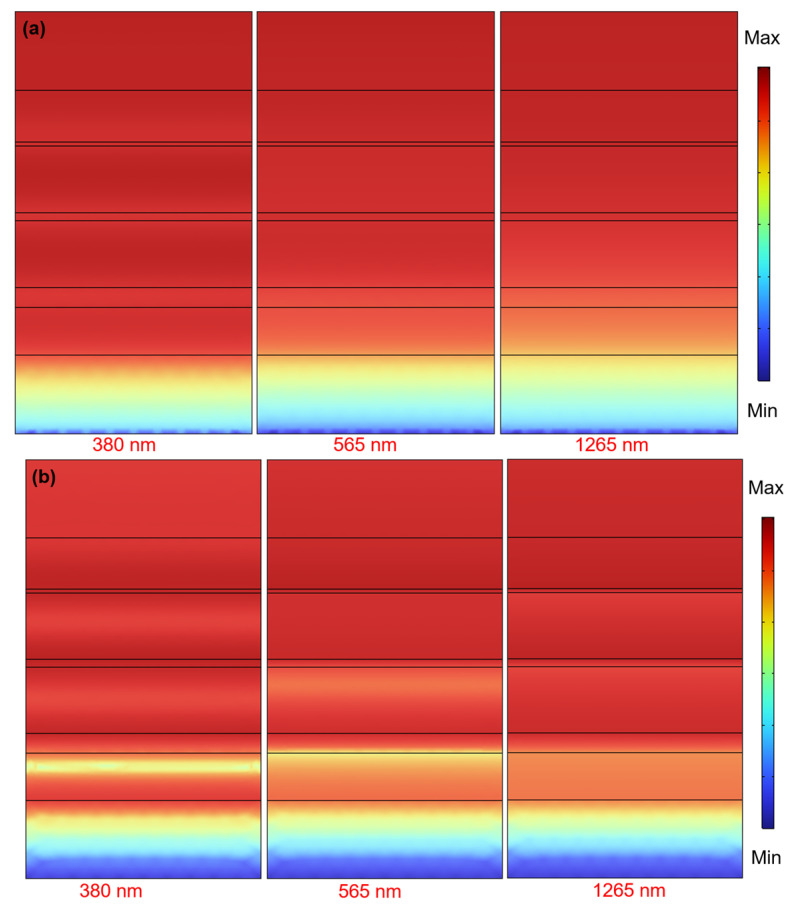
The distributions of (**a**) electric field and (**b**) magnetic field intensities examined under normal incident TE-polarized light with varying exciting wavelengths.

To elucidate the fundamental physical mechanisms driving the high absorptivity and exceptionally wide bandwidth observed in the absorber under investigation, simulations were conducted to analyze the spatial distributions of electric and magnetic field intensities, and Figure 6a,b illustrate these distributions in the x-z plane. The incident light used to excite the absorber was TE-polarized and normally directed, with wavelengths of 380, 565, and 1265 nm. These wavelengths were specifically chosen to align with the absorption peaks identified in Figure 4’s absorption spectrum. These peaks signify resonance phenomena occurring within the studied material or structure. Each absorption peak corresponds to a distinct resonance mode at a particular wavelength, wherein the material or structure exhibit a significant enhancement of either the electric or magnetic field intensities at that specific wavelength. Propagation surface plasmon resonance (PSPR), Fabry–Perot cavities, and localized surface plasmon resonance (LSPR) are pivotal techniques in the production of ultra-thin optical absorption gases, each with their distinct operational principles. PSPR, for instance, revolves around the phenomenon of plasmon resonance initiated on the surface of a metallic thin film.

Under precise conditions of incidence angle and wavelength, a surface plasmon wave, termed the propagation surface plasmon wave, arises at the interface between the incident light wave and the metallic thin film, giving rise to resonance. The control over absorption of specific wavelengths of light in PSPR primarily stems from adjustments in the thickness of the metallic thin film and the refractive index of the dielectric. Notably, PSPR can manifest between different layers, however, its penetration into deeper materials is inherently limited. It is evident that under the conditions of the three wavelengths, the high-energy electric field distribution extends from the first layer all the way to the eighth layer, while the electric field distribution spans from the first layer to either the seventh or eighth layer. Therefore, it is unlikely that PSPR is the cause of the resonances at 380, 565, and 1265 nm. This suggests that other factors or mechanisms may be contributing to the observed resonances at these specific wavelengths.

The Fabry–Perot cavity principle entails an optical resonator formed by two reflective surfaces, typically parallel to each other. When light waves undergo successive reflections within the cavity, significant enhancement occurs at the resonant frequency if the wavelength of the light wave satisfies the resonant conditions of the cavity. Fabry–Perot cavities find common applications in optical filters, lasers, and optical sensors. The absorption of specific frequency light waves is primarily achieved by adjusting the geometric structure and materials of the cavity. Therefore, if the Fabry–Perot cavity effect is to be generated, it mainly occurs within the oxide (dielectric material). However, the electric field distributions (Figure 6a) do not show the occurrence of Fabry–Perot cavity energy distribution, while the electric field distributions (Figure 6b) exhibit indistinct Fabry–Perot cavity energy distribution at 380 and 565 nm. Thus, the Fabry–Perot cavity principle is not the primary cause of the three absorption peaks at 380, 565, and 1265 nm.

LSPR is a phenomenon of plasmon resonance induced on the surface of metallic nanostructures. When the size and shape of the metallic nanostructures match the wavelength of the incident light, surface electrons resonate, forming localized plasmon resonance. This resonance leads to enhanced electric fields near the metal surface, thereby amplifying the interaction between light and matter. The principle behind the generation of different optical absorption peaks in LSPR primarily involves two factors: interlayer spacing and material properties. In multi-layer planar structures, the thickness of each layer and the spacing between layers affect the occurrence of LSPR. LSPR is a phenomenon of interaction between electromagnetic fields and surface plasmon polaritons on a metal surface. When incident light resonates with the metal surface, significant local field enhancement occurs, thereby enhancing optical absorption.

Changes in interlayer spacing lead to variations in resonance frequency, thereby generating different absorption peaks. In addition to interlayer spacing, the properties of the materials used in multi-layer planar structures also play a crucial role in the generation of LSPR. The refractive index and absorption coefficient of both the metal and dielectric layers affect the frequency and intensity of LSPR. Different material combinations result in different LSPR characteristics, thus generating distinct optical absorption peaks. Therefore, the generation of different optical absorption peaks in multi-layer planar structures is the result of the combined effects of interlayer spacing and material properties. By adjusting interlayer spacing and selecting suitable materials, the characteristics of LSPR can be controlled, enabling efficient absorption of light at specific wavelengths. Therefore, it can be observed that the design primarily relies on LSPR for the absorption.

**Figure 7:**
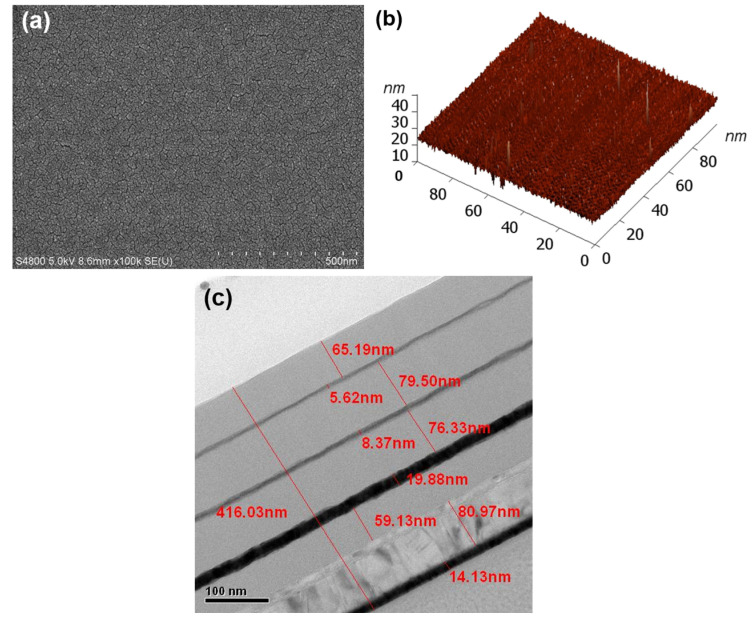
Structure analyses of the fabricated multi-layer planar solar light absorber, (**a**) FESEM observation, (**b**) AFM analysis, and (**c**) TEM observation.

The structure analyses of the fabricated multi-layer planar solar light absorber were conducted through FESEM observation, AFM analysis, and TEM observation, as depicted in Figure 7a, Figure 7b and Figure 7c, respectively. Figure 7a reveals the highly uniform surface morphology of the topmost t1 Al_2_O_3_ layer. In Figure 7a, it is observed that the Al_2_O_3_ thin film deposited by the evaporation method exhibits exceptional uniformity and smoothness. Although only the top Al_2_O_3_ layer is visible, each layer appears to be deposited neatly atop the previous one. This observation provides initial evidence that the evaporation method employed in this study yields high-quality thin films, a notion that will be further substantiated in subsequent analyses. To validate the effectiveness of the evaporation method in fabricating high-performance multi-layer planar solar light absorbers, AFM was employed to analyze the surface of the topmost t1 Al_2_O_3_ layer, with the results presented in Figure 7b. The measured Ra, Sa, and Sq were 0.7048, 0.9933, and 1.4451 nm, respectively. Ra signifies the average roughness of the surface, indicating the average deviation of surface heights within a specified measuring length; Sa represents the surface arithmetic mean, reflecting the average deviation of surface heights across the entire measured area; Sq denotes the root-mean-square roughness, providing insight into the overall fluctuation or undulation of the surface height deviations.

Figure 7c illustrates the results of the TEM analysis conducted on each layer. The thicknesses of the t1-t8 layers and the Ni metal layer were measured at 65.19, 5.62, 79.50, 8.37, 76.33, 19.88, 59.13, 80.97, and 14.13 nm, respectively. Conversely, the optimal simulated thicknesses for the t1-t8 layers and the Ni adhesion metal layer were calculated as 65, 5, 85, 10, 85, 25, 60, 80, and 15 nm. Consequently, the discrepancies between the actual fabricated thicknesses and the optimal simulated thicknesses for the t1-t8 layers and the Ni adhesion metal layer were +0.29, +12.40, −6.47, −16.3, −10.20, −20.48, −1.45, +1.21, and −5.80, respectively. Positive and negative values here indicate whether the deposited thickness is thinner or thicker than the optimal simulated thickness. Nonetheless, the discrepancies in absorber’s thicknesses between the actual fabrication and the optimal simulated values fall within the acceptable ranges.

**Figure 8:**
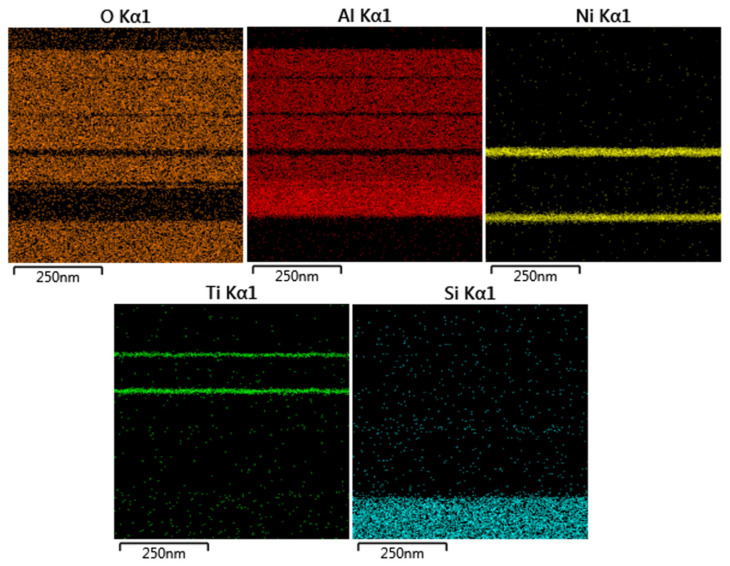
Analyses results of EDS mapping, coupled with TEM observations.

The results obtained from energy dispersive X-ray spectroscopy (EDS) mapping, in conjunction with TEM observations, offer valuable insights into the elemental composition and distribution within the sample under examination. By correlating TEM images with EDS maps, researchers can precisely determine the spatial arrangement of elements at the nanoscale, facilitating the understanding of material properties, chemical variations, and interface structures. This integrated approach serves as a comprehensive characterization tool for studying the microstructure and composition of materials with exceptional spatial resolution and elemental specificity. Based on the EDS analysis results in Figure 8, we clearly observe the distribution of elements such as oxygen, aluminum, nickel, titanium, and silicon, which perfectly aligns with the cross-sectional observation position in Figure 7c. This detailed analysis of element distribution across different regions of the sample provides us with a deeper understanding of the material’s composition and structural characteristics. These findings further confirm the presence of these elements in the studied sample and illustrate their distribution across various regions.

**Figure 9:**
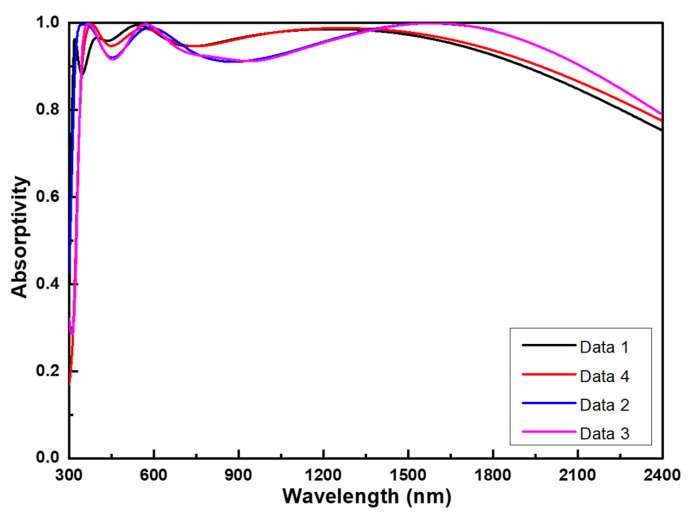
Absorption spectra of the designed multi-layer planar solar light absorber excited by normal incidence. Data 1: the original simulation result; Data 2 and Data 3: two different locations on the identical sample of the fabricated multi-layer planar solar light absorber; Data 4: simulation result obtained by using the TEM measured thicknesses.

To illustrate the correspondence between simulation and implementation, Figure 9 presents comparisons of four distinct result sets. The initial set includes results derived from simulation results utilizing optimal parameters (referred to as Data 1, based on COMSOL simulation data). The second and third sets comprise measurements from two different locations on the identical sample of the fabricated multi-layer planar solar light absorber (referred to as Data 2 and Data 3, respectively, representing actual measurement datasets). The fourth set entails integrating thickness data acquired from Figure 7c into COMSOL simulations (referred to as Data 4, indicating the implementation of simulated data in TEM observation). It is evident that Data 1, Data 2, Data 3, and Data 4 exhibited sustained high absorptivity (>90%) across wideband ranges spanning 346 to 1971 nm, 318 to 2115 nm, 342 to 2118 nm, and 315 to 1914 nm, respectively. These findings imply that Data 1, Data 2, Data 3, and Data 4 possessed a bandwidth (BW) reaching up to 1625 nm, 1797 nm, 1776 nm, and 1599 nm, respectively. Within the high absorptivity (>90%) range, the maximum absorptivity values were recorded as 0.996 (at 384 nm), 0.999 (at 1590 nm), 0.999 (at 1587 nm), and 0.995 (at 546 nm), respectively.

The average absorptivity was calculated using the equation of $A=\int_{\lambda 2}^{\lambda 1}A\left(\lambda \right)d\lambda /\left(\lambda 1-\lambda 2\right)$, where the used λ1 values were 346, 318, 342, and 315 nm and λ2 values were 1914, 2115, 2118, and 1914 nm, which could be 0.965, 0.959, 0.958, and 0.964, respectively. These results demonstrate that both sets of measured data (Data 2 and Data 3) confirm the effectiveness of the designed multi-layer planar solar light absorber in achieving wideband high absorptivity. Notably, the results from both sets of measured data (Data 2 and Data 3) closely align with those obtained when simulating with actual thicknesses (Data 4), as depicted in Figure 7c. One potential factor contributing to the disparities between them could be the variance in deposited thicknesses compared to the optimal simulated values. Another possible reason for these differences might stem from the non-uniformity of the thin film deposition process and the minimal surface roughness observed in the deposited films, as depicted in Figure 7c. Nonetheless, both simulation and implementation can achieve ultra-wideband characteristics, making them suitable for use as solar light absorbers.

**Figure 10:**
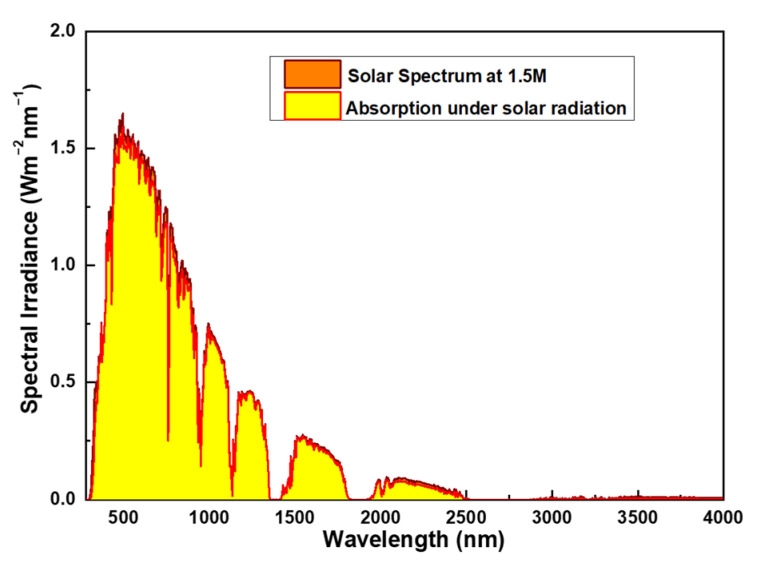
Absorption spectrum of the investigated absorber under the simulation exposed to AM1.5 solar spectrum irradiation.

To explore the absorptive characteristics of the solar energy absorber, it is essential to examine its absorption of solar radiation. Figure 10 illustrates the calculated absorption of solar energy by the simulated absorber across the AM1.5 spectrum (from 300 nm to 4000 nm). A comparison of absorption rates at various wavelength ranges with different data (data 1, data 2, and data 4) presented in the preceding paragraph can be found in Table 1. The absorption spectrum reveals distinct bands spanning from 300 to 4000 nm, including intervals at 300–400 nm, 400–700 nm, 700–1400 nm, 1400–3000 nm, and 3000–4000 nm. The wavelength range exhibiting high absorption corresponds to the high-energy segment of the primary solar radiation. Of particular note is the exceptional absorptivity displayed within specific wavelength bands: data 1, data 2, and data 4 consistently exhibit absorptivity levels exceeding 0.900 of AM1.5G solar energy, highlighting their efficacy across the electromagnetic spectrum. Notably, these data points also showcase significant absorption capacity under the AM1.5 solar spectrum, with absorptivity values of 0.956, 0.962, and 0.948, respectively, across the entire absorption band spanning from 300 to 4000 nm. These results also demonstrate that regardless of the method used to obtain the spectrum, all absorptivity within the range of 300–3000 nm exceeds 0.900, although there may be some differences.

The results from Figure 10 also indicate the absorption range of solar radiation, primarily lying between wavelengths of 300 to 2000 nanometers. This is due to the differential absorption characteristics of the atmosphere for various wavelengths of solar radiation. Within this range, designing absorbers with absorption bandwidths matching this interval allows for efficient absorption of solar energy, encompassing both visible light and infrared radiation. However, once the wavelength exceeds 2000 nanometers, the energy density of solar radiation sharply decreases. Consequently, radiation beyond this range has a relatively minor impact on the absorption efficiency of absorbers. Despite absorbing over 90% only up to 1970 nanometers, it is evident from both practical implementation and simulations that the overall solar absorption ratio (300–4000 nanometers) has already exceeded 94.8%. This proves that the absorber we investigated possesses high absorptivity and ultra-wideband characteristics. These findings underscore the effectiveness of the designed absorber in capturing solar energy across a wide spectrum, encompassing ultraviolet, visible, near-infrared (IR), and mid-IR wavelengths. Moreover, the observed absorption properties suggest nearly ideal wideband absorption throughout the ultraviolet and mid-IR spectrum, which holds considerable promise for energy harvesting initiatives and solar conversion technologies.

## 4. Conclusions

After simulation, the optimized thicknesses for the investigated multi-layer planar solar light absorber were determined: 65 nm for the t1 (Al_2_O_3_) layer, 5 nm t2 (upper Ti) layer, 85 nm for t3 (upper middle Al_2_O_3_) layer, 10 nm for t4 (lower Ti) layer, 85 nm for t5 (lower middle Al_2_O_3_) layer, 25 nm for t6 (Ni) layer, 60 nm for t7 (lower Al_2_O_3_ layer, and 100 nm t8 (Al) layer. The simulation results validated that the high ultra-wideband and high absorptivity are primarily attributed to two key factors: localized surface plasmon resonance and optical impedance matching. The simulation results, employing optimal parameters (Data 1), as well as both sets of measured data (Data 2 and Data 3), along with simulations using actual thicknesses (Data 4), consistently demonstrated high absorptivity levels (>90%) across broad wavelength ranges: 346 to 1971 nm (bandwidth reaching up to 1625 nm), 318 to 2115 nm (1797 nm), 342 to 2118 nm (1776 nm), and 315 to 1914 nm (1599 nm), and the average absorptivity in the wideband range could be 0.965, 0.959, 0.958, and 0.964, respectively. The absorption capacity under the AM1.5 solar spectrum across the entire absorption band spanning from 300 nm to 4000 nm for data 1, data 2, and data 4 had the absorptivity values of 0.956, 0.962, and 0.948, respectively. The proposed ultra-wideband absorber outlined in this paper holds promising potential across various fields, including solar power generation, thermal electronic equipment, and perfect cloaking applications.

While simulations play a crucial role in designing and optimizing optical absorbers, the actual manufacturing of these materials remains challenging. Nanofabrication techniques demand highly precise equipment and control, with even minor variations in process parameters potentially exerting significant impacts on the final product’s performance. This is particularly evident in cases where thicknesses are as minute as 5 nm, pushing the boundaries of proposed solutions. Despite the successful deposition of nanoscale films in this study, deviations in film thickness persisted. Therefore, future endeavors, beyond attempting to design optical absorbers with broader bandwidths, must involve seeking collaborative partners capable of depositing films with greater precision. This collaboration aims to enhance coating results after the efficient overall structure has been designed.

## Figures and Tables

**Table 1:** Absorptivity of the designed absorber under the different wavelength ranges of the AM1.5 solar spectrum. Original (data 1): the original simulation result; Measurement 1 (data 2): result from the first measurement; Real thickness (data 4): simulation result obtained by using the TEM measured thicknesses.

	Absorptivity of AM 1.5G Solar Energy		
Wavelength (nm)	Data 1	Data 2	Data 4
300~400	0.908	0.933	0.977
400~700	0.958	0.973	0.960
700~1400	0.966	0.966	0.931
1400~3000	0.924	0.913	0.955
3000~4000	0.440	0.431	0.468
Total (280~4000)	0.956	0.962	0.948

## Data Availability

Data are contained within the article.
